# Evidence-based clinical practice guidelines for Liver Cirrhosis 2020

**DOI:** 10.1007/s00535-021-01788-x

**Published:** 2021-07-07

**Authors:** Hitoshi Yoshiji, Sumiko Nagoshi, Takemi Akahane, Yoshinari Asaoka, Yoshiyuki Ueno, Koji Ogawa, Takumi Kawaguchi, Masayuki Kurosaki, Isao Sakaida, Masahito Shimizu, Makiko Taniai, Shuji Terai, Hiroki Nishikawa, Yoichi Hiasa, Hisashi Hidaka, Hiroto Miwa, Kazuaki Chayama, Nobuyuki Enomoto, Tooru Shimosegawa, Tetsuo Takehara, Kazuhiko Koike

**Affiliations:** 1 Guidelines Committee for Creating and Evaluating the “Evidence-Based Clinical Practice Guidelines for Liver Cirrhosis”, The Japanese Society of Gastroenterology / The Japan Society of Hepatology, 6F Shimbashi i-MARK Building, 2-6-2 Shimbashi, Minato-ku, Tokyo, 105-0004 Japan; 2 The Japan Society of Hepatology, Kashiwaya 2 Building 5F, 3-28-10 Hongo, Bunkyo-ku, Tokyo, 113-0033 Japan; 3 Department of Gastroenterology, Nara Medical University, Shijo-cho 840, Kashihara, Nara 634-8522 Japan

**Keywords:** Liver cirrhosis, Guidelines, Diagnosis, Treatment, Complications

## Abstract

The first edition of the clinical practice guidelines for liver cirrhosis was published in 2010, and the second edition was published in 2015 by the Japanese Society of Gastroenterology (JSGE). The revised third edition was recently published in 2020. This version has become a joint guideline by the JSGE and the Japan Society of Hepatology (JSH). In addition to the clinical questions (CQs), background questions (BQs) are new items for basic clinical knowledge, and future research questions (FRQs) are newly added clinically important items. Concerning the clinical treatment of liver cirrhosis, new findings have been reported over the past 5 years since the second edition. In this revision, we decided to match the international standards as much as possible by referring to the latest international guidelines. Newly developed agents for various complications have also made great progress. In comparison with the latest global guidelines, such as the European Association for the Study of the Liver (EASL) and American Association for the Study of Liver Diseases (AASLD), we are introducing data based on the evidence for clinical practice in Japan. The flowchart for nutrition therapy was reviewed to be useful for daily medical care by referring to overseas guidelines. We also explain several clinically important items that have recently received focus and were not mentioned in the last editions. This digest version describes the issues related to the management of liver cirrhosis and several complications in clinical practice. The content begins with a diagnostic algorithm, the revised flowchart for nutritional therapy, and refracted ascites, which are of great importance to patients with cirrhosis. In addition to the updated antiviral therapy for hepatitis B and C liver cirrhosis, the latest treatments for non-viral cirrhosis, such as alcoholic steatohepatitis/non-alcoholic steatohepatitis (ASH/NASH) and autoimmune-related cirrhosis, are also described. It also covers the latest evidence regarding the diagnosis and treatment of liver cirrhosis complications, namely gastrointestinal bleeding, ascites, hepatorenal syndrome and acute kidney injury, hepatic encephalopathy, portal thrombus, sarcopenia, muscle cramp, thrombocytopenia, pruritus, hepatopulmonary syndrome, portopulmonary hypertension, and vitamin D deficiency, including BQ, CQ and FRQ. Finally, this guideline covers prognosis prediction and liver transplantation, especially focusing on several new findings since the last version. Since this revision is a joint guideline by both societies, the same content is published simultaneously in the official English journal of JSGE and JSH.

## Introduction

Cirrhosis is a terminal image of chronic liver disease. During progression from the compensation period to the decompensation period, various complications occur and the life prognosis is significantly reduced. In recent years, medical treatment for liver cirrhosis has made marked progress that can be said to be a paradigm shift.

In recent years, the management of liver diseases has undergone major changes. For example, antiviral therapy for hepatitis C virus (HCV) has changed significantly due to the advent of novel oral direct antiviral drugs (DAA), but the management of background liver disease, mainly liver cirrhosis, remains a clinically important issue even after HCV eradication. DAA has recently been approved even for patients with decompensated cirrhosis and is expected to contribute to the improvement of the prognosis of patients with cirrhosis. However, not all cases show improvement in hepatic reserve, and whether portal pressure improves after DAA treatment is still a controversial issue. Since there are several cases in which portal pressure gets worse after HCV eradication, the research on “point of no return” still remains a major issue in clinical practice. In addition to HCV, many new findings have been reported in the last 5 years regarding the treatment of hepatitis B liver cirrhosis with newly developed nucleic acid analogs. In addition, the etiology of cirrhosis has changed significantly over time, and several recent studies have reported that non-viral-mediated cirrhosis has significantly increased over the last decade. It has been reported that the incidence of liver cancer in patients with non-viral cirrhosis is much lower than that in patients with viral cirrhosis. In addition to carcinogenesis suppression, which has been the most clinically significant issue in patients with cirrhosis to date, it is expected that the treatment of various complications based on an understanding of the pathological condition will become more clinically important in the near future.

Regarding cirrhosis, not only a single disease but also various causes, such as hepatitis viruses, alcohol, fatty liver, and many complications, including the whole disease state, are closely related to liver cancer. The CQ number was over 80 in the second edition [1]. The revised guidelines by the Japanese Society of Gastroenterology suggest that the number of CQs be reduced to 20 to 30. However, for cirrhosis, it was difficult to significantly reduce the CQ number because new findings regarding the condition of cirrhosis treatment have been reported one after another over the past 5 years since the second edition, and the concept itself has changed significantly, such as the inclusion of hepatorenal syndrome. In addition, a number of new therapeutic agents for various complications associated with cirrhosis have been launched since the second edition, and CQ and FRQ are required for these new agents to be useful in clinical practice. Therefore, many clinically important issues that were CQs are now BQs. In addition, as mentioned above, many new drugs have been developed in the past 5 years, and due to the current multiple clinical trials in progress, new treatments that are clinically important in the future will be newly identified under FRQ. Newly added items include treatment with an oral DAA for type C decompensated cirrhosis, sarcopenia, muscle cramp, pruritus, hepatopulmonary syndrome, pulmonary hypertension associated with portal hypertension (PoPH), vitamin D deficiency, which has been shown to be involved in the pathological conditions of liver cirrhosis, and alcohol reduction therapy (harm reduction) for liver cirrhosis.

The editorial chairman of this revision is H. Yoshiji, and the deputy editorial chairman is S. Nagoshi; experts in each field contributed to the writing This revision was performed according to the Minds clinical practice guideline preparation manual, and the preparation was advanced so that it would be the second edition. CQs and FRQs were collected through published papers by extracting keywords. CQs and FRQs were searched for in English articles in MEDLINE and Cochrane Library [2] and in Japanese articles in the Japan Medical Library Association, focusing on the Central Medical Journal. Regarding the BQs, a hand search was carried out by each preparation committee. The CQ and FRQ literature search period was set between October 1983 and October 2018 for English literature and between the year 1983 and December 2018 for Japanese literature. After-document retrieval period, required papers are also added at the discretion of the preparation committee members to reflect the latest evidence. Of the collected papers, clinical studies conducted on humans were adopted, and papers on animal experiments were excluded in principle. We also referred to the opinions of individual experts based on patient data but did not use them as evidence. A structured abstract was prepared for necessary issues, such as new CQs and FRQs, for this revision because searching had been sufficiently performed for the second edition of 2015. The quality of the evidence was rated on a four-point scale from A to D. Grade A is high-quality evidence, B is medium-quality evidence, C is low-quality evidence, and D is very low-quality evidence. The strength of the recommendation consisted of four items: 1: certainty of evidence (strength), 2: patient’s wishes, 3: benefits and harms, and 4: cost evaluation. There are two strengths of recommendation, strong and weak, and the voting result is described as the consensus rate. After drafting the preparation committee, each CQ was voted on by all members for the recommendation grade decision [3–12]. Consensus was previously defined as 70% or more votes in agreement. After that, the revisions were repeated by the preparation committee, and the final proposal was consulted with the evaluation committee. The revisions were repeated, and the final proposal was consulted with the evaluation committee. After reviewing the report of the evaluation committee and making revisions, public comments were posted on the websites of the Japanese Society of Gastroenterology and Japan Society of Hepatology, and solicited. After the public comments, the revised plan was confirmed, and the final revision was worked on by each preparation committee member to complete the revised third edition.

This guideline is based on evidence up to 2019 regarding the treatment of liver cirrhosis and supports clinical practice by presenting the contents of medical treatment that can be recommended and proposed. The disease states of patients with cirrhosis are extremely diverse, and some medical treatment methods are outside the scope of insurance medical treatment. Please note these points when applying this guideline and take appropriate measures, such as avoiding use for purposes other than medical treatment.

## Diagnosis and etiology

### **BQ1-1**. What is the etiology and pathogenesis of liver cirrhosis?

- Liver cirrhosis is established by the process of necrosis and regeneration of hepatocytes, resulting in fibrosis and capillarization of the hepatic sinusoid. Decreased hepatic parenchyma, disturbance of blood flow due to fibrosis and abnormal reconstruction, and portosystemic shunt cause portal hypertension; ascites; hepatic encephalopathy; pulmonary, renal, and cardiac disturbance; and hyponatremia. Liver cirrhosis presents a high risk for the development of hepatocellular carcinoma. Hepatitis C virus (HCV) has remained the main cause of liver cirrhosis in Japan. However, the contribution of HCV as an etiology of liver cirrhosis has been decreasing. Non-viral Liver cirrhosis, such as alcoholic-related disease (ALD) and nonalcoholic steatohepatitis (NASH), has increased as etiologies of liver cirrhosis in Japan [13].

### **FRQ 1–1**. What is the pathogenesis of acute-on-chronic liver failure (ACLF)?

Statement:

- ACLF is an acute decompensation stage in patients with liver cirrhosis, which develops and worsens because of some triggers and leads to liver failure within 28 days [14]. Although the exact pathogenesis of ACLF progression is not known, it requires multidisciplinary treatment for multiple organ failure and has a high mortality rate [15]. In Japan, the proposal for the diagnostic criteria for ACLF has been confirmed [16], and nationwide studies will be performed to elucidate the pathogenesis, prognosis, and therapy for ACLF.

## Diagnosis of liver cirrhosis

### **BQ 2–1**. Are biochemical examination of blood and imaging findings useful for the diagnosis of liver cirrhosis?

- The determination of fibrosis scores based on several blood tests and evaluation of liver stiffness by elastography is useful for the diagnosis of liver cirrhosis.

**Fig. 1:**
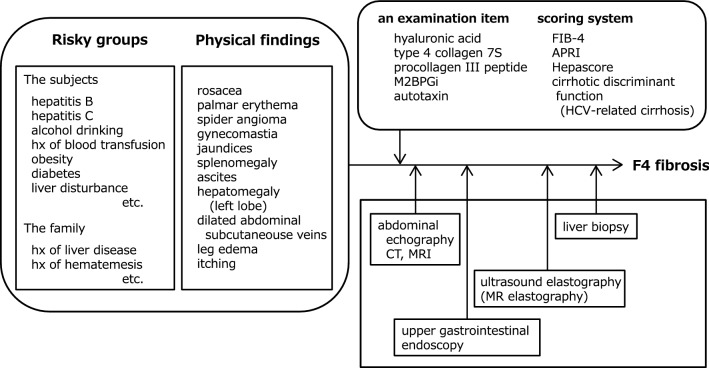
Diagnostic algorithm for liver cirrhosis. After obtaining basic information on the cause of liver cirrhosis, characteristics of the patients, and physical examinations, we should combine several diagnostic tools, such as serum biomarkers, imaging modalities, and endoscopy, as noninvasive alternatives to liver biopsy. This algorithm diagnoses cirrhotic F4 fibrosis. A special blood test or histological characteristics are often needed to determine the cause of cirrhosis. *APRI* aspartate aminotransferase to platelet ratio index, *FIB-4* fibrosis-4 index, *HCV* hepatitis C virus, *hx* history, *M2BPGi* Mac-2 binding protein glycosylation isomer

A diagnostic algorithm is shown in Fig. 1.

## Nutritional therapy

### **BQ3-1**. Do under-nutrition and obesity affect the prognosis of patients with cirrhosis?

- Appropriate measures, such as nutritional therapy, are required because under-nutrition and obesity in patients with cirrhosis affect their prognosis.

### **BQ3-2**. Does a late-evening snack (LES) affect the pathological condition of patients with liver cirrhosis?

- An LES improves the pathological condition of patients with liver cirrhosis.

### **BQ3-3**. Is the administration of branched-chain amino acids (BCAAs) effective in improving the pathogenesis of liver cirrhosis?

- BCAAs should be administered if patients with cirrhosis present with protein energy malnutrition.

### **BQ3-4**. What is the recommended energy/protein intake for patients with cirrhosis?

- Energy intake is based on 25–35 kcal/kg (standard body weight)/day in the absence of glucose intolerance, and protein requirements are based on 1.0–1.5 g/kg/day (including BCAA preparations) in the absence of protein intolerance.

**Fig. 2:**
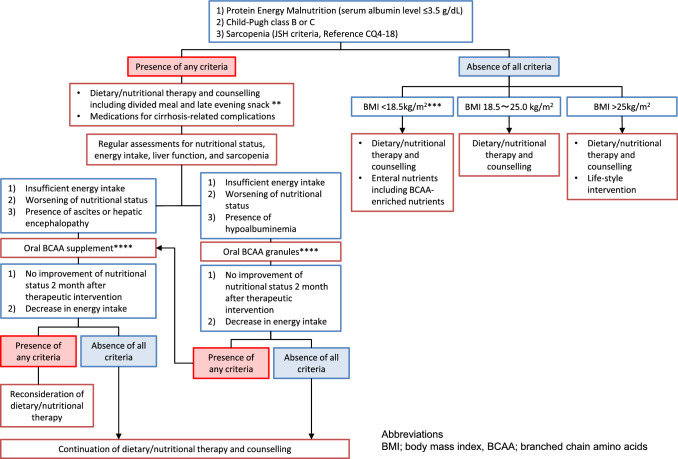
Nutritional therapy. *There is no gold standard for the assessment of nutritional status. It is recommended to comprehensively evaluate nutritional status by assessing dietary intake, body composition, and biochemical examinations. Measurement of non-protein respiratory quotient (npRQ) using indirect calorimetry is recommended for the assessment of energy malnutrition. However, the measurement of npRQ is limited in general medical practice. In patients with liver cirrhosis, %arm circumference (%AC) < 95 and fasting plasma free fatty acid (FFA) levels > 660 μEq/L are indices to predict npRQ < 0.85, which is a prognostic marker. Changes in fasting plasma FFA levels are useful for the dynamic assessment of nutritional status after dietary/nutritional intervention. However, the measurement of plasma FFA levels is not covered by the National Health Insurance of the Japanese Ministry of Health. For patients undergoing invasive treatment for hepatocellular carcinoma or esophagogastric varices, dietary/nutritional therapy is strongly recommended to improve protein-energy malnutrition. The diagnosis of sarcopenia is based on the Japan Society of Hepatology guidelines for sarcopenia in liver disease (Reference, CQ4-18). Measurement of grip strength is used to assess muscle strength. Muscle mass is assessed by skeletal muscle index based on bioelectrical impedance analyzer (BIA) or computed tomography (CT) scans at the lower border of the third lumbar vertebra (L3). Each assessment has advantages and disadvantages. **Based on required daily energy intake, it is recommended to treat cirrhotic patients by dietary/nutritional therapy, including divided meals (3–5 times/day), late evening snack, and BCAA-enriched enteral nutrients. Regular assessments of nutritional status are recommended. In patients with no improvement in nutritional status or energy intake, an oral BCAA supplement is recommended for patients with ascites or hepatic encephalopathy. Oral BCAA granules are recommended for patients with hypoalbuminemia. ***Patients with BMI < 18.5 kg/m^2^ are at high risk of protein-energy malnutrition or sarcopenia. It is recommended to perform regular nutritional assessments and dietary/nutritional intervention using enteral nutrients, including BCAA-enriched nutrients. ****Oral BCAA supplement is approved for “an improvement in the nutritional status of patients with chronic hepatic failure and hepatic encephalopathy.” Oral BCAA granules are approved for “an improvement in hypoalbuminemia in decompensated cirrhotic patients with sufficient energy intake and hypoalbuminemia.” Hypoalbuminemia is defined as a serum albumin level <3.5 g/dL. To prevent disease progression, it is recommended to change oral BCAA granules to alternative treatments in patients with no improvement of hypoalbuminemia 2 months after treatment with oral BCAA granules or no improvement of energy intake or BCAA to tyrosine ratio (BTR) 1 month after treatment with oral BCAA granules

Figure 2 shows the algorithm for nutritional therapy in patients with cirrhosis.

### **CQ3-1**. Does diabetes affect the pathogenesis of cirrhosis?

- Diabetes mellitus and abnormal glucose metabolism have a negative effect on the pathophysiology of liver cirrhosis, such as exacerbation of complications and liver carcinogenesis; therefore, appropriate management and intervention are recommended.

(Recommendation: strong, 100% agreed, evidence level A).

*Comment*: Diabetes mellitus and abnormal glucose metabolism, such as insulin resistance, which are common in patients with liver cirrhosis, increase the risk of cirrhosis complications and liver carcinogenesis [17, 18]. The anti-diabetic drug metformin can be expected to improve the prognosis of patients with decompensated liver cirrhosis and suppress liver carcinogenesis [19, 20]; however, the use of these and other diabetes drugs for decompensated liver cirrhosis requires extreme caution. It is also not clear whether the improvement of abnormal glucose metabolism by anti-diabetic drugs directly contributes to improved prognosis.

### **CQ3-2**. Do divided meals and eating habits affect the pathology of patients with cirrhosis?

- Divided meals and LES are recommended for patients with cirrhosis.

(Recommendation: strong, 100% agreed, evidence level B).

*Comment*: Energy metabolism in patients with cirrhosis is in a hyper-catabolic state. Divided meals (four to seven times per day) are reported to improve non-protein respiratory quotient compared to two meals per day in patients with cirrhosis [21]. Lifestyle interventions, such as diet and exercise, for patients with chronic liver disease have been reported to improve insulin resistance, hepatic steatosis, and liver fibrosis [22]. Meta-analyses also report that daily coffee consumption is associated with the prevention of liver fibrosis development and reduced risk of hepatocellular carcinoma development and death [23, 24].

## Antiviral therapy

### Hepatitis B

#### **BQ3-5**. What hepatitis B virus (HBV)-associated markers are predictive of the prognosis?

- HBV DNA level is a marker of prognosis and development of hepatocellular carcinoma (HCC). The core-related antigen is a marker of HCC development in the natural course and during nucleos(t)ide analog therapy.

#### **CQ3-3**. Is nucleos(t)ide analog therapy for HBV effective for patients with cirrhosis?

- Nucleos(t)ide analog therapy improves liver fibrosis and liver function and inhibits the development of HCC. Therefore, nucleos(t)ide analog therapy is recommended in patients with decompensated and compensated cirrhosis.

(Recommendation: strong, 100% agreed, evidence level A).

*Comment*: Lamivudine [25], entecavir [26], and tenofovir (TDF) [27] have been reported to improve liver fibrosis. A randomized controlled trial (RCT) showed a significantly lower exacerbation of Child–Pugh scores with lamivudine compared to that with placebo [28]. A meta-analysis reported a reduced risk of decompensation and all-cause mortality by nucleos(t)ide analog therapy [29]. Patients who underwent nucleos(t)ide analog therapy for decompensated cirrhosis showed improved transplant-free survival compared to untreated patients [30]. In a prospective observational study of decompensated cirrhosis treated with a nucleos(t)ide analog, improvement in Child–Pugh A after 12 months was 66% with entecavir [31] and 68.4% with tenofovir (TDF) [32]. A meta-analysis of ten papers that analyzed HCC development in cirrhosis reported a significant reduction in HCC incidence by nucleos(t)ide analog therapy [29]. In summary, nucleos(t)ide analog therapy for compensated cirrhosis ameliorates liver fibrosis, prevents hepatic function exacerbation and decompensation, inhibits HCC development, and improves survival. It also improves liver function and improves life expectancy in decompensated cirrhosis.

### Hepatitis C

#### **BQ3-6**. Does fibrosis improve in patients with cirrhosis with a sustained virological response (SVR)?

- Liver fibrosis improves when SVR is obtained.

#### **CQ3-4**. Is surveillance for HCC recommended for patients with cirrhosis who achieved SVR?

- Surveillance using imaging and serum tumor marker measurement is recommended for patients with cirrhosis who achieved SVR.

(Recommendation: strong, 100% agreed, evidence level A).

*Comment*: In patients with cirrhosis with ongoing HCV infection, the annual incidence of HCC was high [33]. Among patients who achieved SVR by interferon therapy, the incidence of HCC was decreased by a hazard ratio (HR) of 0.24 in a pooled analysis of 12 studies [34]. A meta-analysis of 6 studies, including 2,649 cases with advanced liver fibrosis, also revealed a reduction in HCC development with an HR of 0.24 [35]. In a study based on a large U.S. veteran population, the incidence of HCC after SVR in patients with cirrhosis was 1.39% per year [36]. The incidence of HCC after SVR is not different between patients treated with interferon and DAA after adjustment of backgrounds. The annual incidence of HCC is reported to be 1.8–2.5% in cases of cirrhosis in which SVR was achieved by DAA [37–42]. Although the risk of HCC development is reduced by 2.5- to fivefold by SVR, the annual incidence is still high enough to continue surveillance long after achieving SVR.

In addition to surveillance using imaging, the concomitant use of serum tumor markers, such as alpha-fetoprotein (AFP), has been controversial because serum AFP could be influenced by the degree of necroinflammation in the liver. However, the specificity of AFP as a tumor marker is expected to improve after SVR because AFP production from non-cancer sources is reduced with improvement in inflammation. Therefore, surveillance using imaging and serum tumor marker measurement is recommended for patients with cirrhosis who achieved SVR.

#### **CQ3-5**. For which patients with cirrhosis is DAA recommended?

- DAA is recommended for patients with cirrhosis, except in cases of poor prognosis.

(Recommendation: strong, 100% agreed, evidence level A).

*Comment*: The goal of antiviral treatment for cirrhosis is to suppress progression to liver failure and development of HCC and to prolong prognosis by reduction of liver inflammation and fibrosis, followed by elimination of HCV. Recent advances in DAA therapy have enabled us to achieve SVR even in decompensated cirrhosis. The only approved regimen for decompensated cirrhosis in Japan is sofosbuvir in combination with velpatasvir [43]. The safety and efficacy of this regimen in patients with a Child–Pugh score of 13 or more are yet to be confirmed in real-world clinical practice since they were excluded in the phase 3 study. Therefore, it is recommended that treatment of decompensated cirrhosis should be carefully performed with hepatology experts.

A prospective cohort study of approximately 10,000 patients with chronic hepatitis C disease (median follow-up, 33 months) reported a significantly reduced risk of hepatocarcinogenesis and death at 3 years in the DAA-treated group compared to that in the non-DAA-treated group. In a sub-analysis limited to cirrhosis, the inhibitory effect was more pronounced [44]. Therefore, DAAs are useful for the treatment of cirrhosis and are recommended unless the prognosis does not improve due to severe liver impairment or comorbidities.

### Antifibrotic therapy

#### **CQ3-6**. Is there any antifibrotic therapy for viral cirrhosis except antiviral therapy?

- No therapy has confirmed efficiency, but the administration of angiotensin-converting enzyme (ACE) inhibitors or angiotensin II receptor blockers (ARBs) might be considered in patients with compensated liver cirrhosis.

(Evidence level C)

*Comment*: In some patients with decompensated state of liver cirrhosis, the progression of fibrosis cannot be prevented by antiviral therapy; therefore, liver failure occurs. Antifibrotic therapies for decompensated liver cirrhosis have been investigated in clinical trials, but there are no insured treatments for fibrosis at present.

A systematic review reported that the fibrosis score (Ishak or METAVIR) was significantly lower in the ACE inhibitor- or ARB-treated group than in the target group [45]. However, in this study, the seven analyzed articles were not limited to viral hepatitis, and only two articles were analyzed by RCTs; therefore, there is insufficient evidence that ACE inhibitors or ARBs improve fibrosis in viral hepatitis. By contrast, because of the risk of worsening renal function, ACE inhibitors or ARBs are not recommended in patients with ascites in decompensated cirrhosis [46].

### Therapy for nonviral liver cirrhosis

#### **BQ3-7**. Does abstinence from alcohol improve the fibrosis and prognosis of alcoholic cirrhosis?

- Long-term abstinence from alcohol improves the prognosis of alcoholic cirrhosis.

#### **CQ3-7**. Does corticosteroid therapy relieve liver fibrosis and improve the prognosis of patients with autoimmune hepatitis and liver cirrhosis?

- Corticosteroid therapy is proposed for patients with active autoimmune-hepatitis-related cirrhosis because relief of fibrosis and improvement of prognosis are expected for responders.

(Recommendation: weak, 100% agreed, evidence level B).

*Comment*: In patients with active autoimmune-hepatitis-related cirrhosis, treatment should be initiated as this represents a negative prognostic predictor [47]. The frequency of histological cirrhosis decreased from 16 to 11% in 87 patients receiving corticosteroid therapy [48]. Cirrhosis, extensive fibrosis, or both disappeared in eight responder patients [49]. Of the 64 corticosteroid-treated patients with decompensated cirrhosis, 40 patients reverted to a compensated state, and survival was greater compared to that of untreated patients [50]. By contrast, corticosteroid therapy is not indicated for patients with inactive cirrhosis who receive no benefit from the therapy and have an increased risk of drug-induced side effects [51].

#### **CQ3-8**. Does medication improve the fibrosis and prognosis of primary biliary cholangitis (PBC)-related cirrhosis?

Administration of ursodeoxycholic acid (UDCA) has been suggested for PBC-related cirrhosis because of its potential to improve prognosis.

(Recommendation: weak, 100% agreed, evidence level B).

*Comment*: Considering the potential to improve biochemical data and safety, UDCA is suggested as a treatment for PBC. European and American guidelines recommend a dose of 13 to 15 mg/kg of body weight per day [52, 53]. In Japan, the guideline states that it is important to administer a daily dose of at least 600 mg to adults, with a maximum dose of 900 mg.

As concomitant or alternative drugs for UDCA, bezafibrate is expected to improve UDCA refractory cases. An RCT of bezafibrate added to UDCA showed significant improvement in biochemical data at 24 months of treatment [54]. According to the Japanese PBC practice guidelines, concomitant use of bezafibrate may be considered for patients who do not respond to UDCA, especially those whose bilirubin or albumin levels are within the reference range [55].

Concerning corticosteroids for PBC, few 3-year RCTs (19 vs. 17) showed improvements in ALP, protein levels, anti-mitochondrial antibody (AMA) titers, and histopathological progression, but showed no improvement in mortality with a higher rate of non-hepatic adverse complications, such as infections, diabetes, and ulcers [56]. It is not considered a recommended treatment except in cases of suspected PBC-AIH overlap syndrome.

#### **CQ3-9**. Does medication improve the fibrosis and prognosis of primary sclerosing cholangitis (PSC)-related cirrhosis?

- Administration of ursodeoxycholic acid (UDCA) can be considered in individual cases, with the understanding that its efficacy for PSC-related cirrhosis has not been determined yet.
  (Evidence level C)

Corticosteroids do not improve the prognosis of PSC-related cirrhosis and are recommended not to be administered.

(Recommendation: strong, 100% agreed, evidence level A).

*Comment*: As there was no evidence of an improvement in prognosis, there are some debates in other guidelines regarding the use of UDCA for PSC [57–59]. In Japan, patients are often administered UDCA and followed up, and some reports have shown improvement in the biochemical data. UDCA has been shown to improve prognosis in patients with reduced ALP [60] and has not been reported to worsen prognosis at the standard dose of 13–15 mg/kg/day. Based on the abovementioned findings, it is reasonable to consider UDCA as the first-line drug of choice in clinical practice in Japan.

Other agents, such as immunosuppressive drugs, including budesonide and azathioprine, and antibiotics, including vancomycin and metronidazole, have been tried, but their clinical effectiveness remains to be determined. According to a meta-analysis, the use of corticosteroids resulted in strong side effects, no improvement in cholangiopathy, and no significant benefit for patients with PSC [61]. Unless the possibility of IgG4-related sclerosing cholangitis cannot be ruled out, it is recommended not to administer corticosteroids.

Liver transplantation is considered in severe cases, as there is no medical treatment with proven efficacy. However, the results of a national study are not excellent, showing a 5-year survival rate of 78%, a 5-year graft survival rate of 74%, and a 5-year recurrence-free survival rate of 57% in an analysis of 114 patients who underwent living liver transplantation [62].

#### **FRQ3-1**. Are there any treatments for alcoholic cirrhosis other than abstinence?

- Harm reduction through the reduction of alcohol consumption has been proposed, but its usefulness in patients with Liver cirrhosis should be examined in future studies.

#### **FRQ3-2**. Is there any pharmacotherapy that improves hepatic fibrosis in patients with non-alcoholic steatohepatitis (NASH)-related liver cirrhosis?

- There is currently no pharmacotherapy that can improve hepatic fibrosis in patients with NASH-related liver cirrhosis.

## Esophagogastric bleeding and portal hypertension

### **BQ4-1**. Are red color signs (RC signs) on upper endoscopy a risk factor for esophageal and gastric variceal bleeding?

- RC signs on upper endoscopy are one of the risk factors for esophageal and gastric variceal bleeding.

### **BQ4-2**. Are abdominal ultrasonography, abdominal contrast-enhanced computed tomography (CE-CT), and magnetic resonance imaging (MRI) useful for the diagnosis of portal hypertension?

- These imaging modalities are useful for the diagnosis of portal hypertension.

### **CQ4-1**. What drugs are useful to prevent bleeding from esophagogastric varices?

- Nonselective beta-blockers, isosorbide mononitrate, or a combination of both drugs are proposed for the prevention of esophagogastric variceal bleeding.

(Recommendation: weak, 100% agreed, evidence level B).

*Comment*: Nonselective beta-blockers (NSBBs̄) improve portal hypotension. Propranolol and nadolol have been used, and evidence of their efficacy has been reported [63–71]. A recent meta-analysis showed significant prevention of variceal bleeding in patients with ≥ 10% reduction of hepatic venous pressure gradient (HVPG) by NSBBs [72]. Recently, carvedilol, an alpha- and beta-blocker, has received attention as a more potent drug to reduce HVPG. In an RCT of carvedilol and propranolol, the number of patients who had reduced HVPG at 1 month after treatment with carvedilol was significantly higher compared to that of patients using propranolol [73]. A meta-analysis using Cochrane and other databases validated that carvedilol was more effective in reducing HVPG; however, there were no significant differences in prognosis, gastrointestinal bleeding, or adverse events between carvedilol and classic NSBBs [74]. Isosorbide-5-mononitrate (IsMn) increases NO and lowers intrahepatic vascular resistance. It decreases the HVPG, azygous venous blood flow, and venous pressure of varices, and has little effect on blood pressure [75]. A meta-analysis of 18 RCTs reported in 2011 indicated that both endoscopic esophageal variceal ligation (EVL) and NSBBs are useful to prevent initial bleeding in esophageal varices, and the combination of NSBB and IsMn has been recommended as the best treatment to prevent rebleeding from esophagogastric varices [76].

### **CQ4-2**. Are vasoactive agents effective for the management of esophagogastric variceal bleeding?

- Vasoactive agents, such as terlipressin and octreotide, have been proposed to be used to control esophageal variceal bleeding.

(Recommendation: weak, 100% agreed, evidence level B).

*Comment*: Terlipressin, a V1 receptor agonist, constricts the abdominal visceral artery and reduces portal blood flow. Terlipressin is frequently used in Europe, and in a meta-analysis of 30 RCTs published in 2018, terlipressin treatment significantly controlled bleeding within 48 h and reduced in-hospital mortality [77]. In comparison to vasopressin, terlipressin had significantly fewer complications. Somatostatin and its analogue, octreotide, also reduce portal hypertension, with a mechanism of inhibition of glucagon secretion and a direct effect on vascular smooth muscles [78]. Octreotide inhibits the postprandial increase in portal blood pressure that cannot be controlled by propranolol; however, its effect is transient and unsuitable for long-term management of portal pressure [79].

In a meta-analysis examining whether vasoactive agents (somatostatin, terlipressin, vapreotide, octreotide) could reduce the risk of death in cases of esophageal variceal bleeding, the use of vasoactive agents significantly reduced the risk of all-cause mortality and number of blood transfusions and shortened the period of hospitalization [80]. In addition, comparative studies among vasoactive agents have not found differences in efficacy. The 2018 meta-analysis also compared terlipressin, somatostatin, and octreotide and indicated that, although terlipressin had significantly fewer complications than somatostatin, terlipressin was significantly inferior to octreotide in controlling esophageal variceal bleeding within 24 h [77].

### **CQ4-3**. What drugs are useful for the management of portal hypertensive gastropathy (PHG)?

- Nonselective beta-blockers (NSBBs) have been proposed to be used for the management of PHG.
  (Recommendation: weak, 100% agreed, evidence level B)

*Comment*: PHG is a congestive gastric mucosal condition caused by portal hypertension, which results in acute gastric bleeding. The treatment is a nonselective beta-blocker (NSBB), especially, propranolol, which has been widely studied. Propranolol significantly reduces portal venous pressure and the rate of rebleeding from PHG [81]. An RCT in 2001 indicated a lower incidence of PHG after EVL in the propranolol group than in the non-treated group [82]. The RCT for patients with gastric varices indicated for EVL showed that PHG was increased significantly 1 year after EVL treatment; however, in the propranolol and carvedilol groups, a significant decrease in PHG was observed [83]. There was no difference in treatment effect between the two NSBB groups; however, adverse events were fewer in the carvedilol group than in the propranolol group.

The use of IsMn in combination with nadolol has been reported; however, any benefit in the reduction of development of PHGs was not evident [84]. Although there are some RCTs using somatostatin for the treatment of acute hemorrhage from PHG, there are no meta-analyses, and the evidence for octreotide is not sufficient.

Several RCTs have reported the benefit of NSBBs in preventing bleeding from PHG; however, there is no meta-analysis. Moreover, the evidence on carvedilol is insufficient because most of the previous reports used propranolol.

### **CQ4-4**. Are acid suppressing drugs useful for preventing gastrointestinal bleeding in patients with cirrhosis?

- Short-term administration of acid suppression therapy is proposed for preventing the recurrence of esophagogastric varices (EV).

(Recommendation: weak, 100% agreed, evidence level B).

*Comment*: There is no data indicating that acid suppression is useful for the prevention of intestinal bleeding in patients with cirrhosis [85]. However, two studies showed that acid suppression therapy was effective for preventing the recurrence of EV ulcer bleeding in patients with cirrhosis [86, 87]. On the contrary, a meta-analysis showed that long-term administration of acid suppression therapy increased the occurrence rate of spontaneous bacterial peritonitis (SBP) [88]. One-year acid suppression treatment significantly worsened hepatic encephalopathy [89]. Moreover, 2.7 years of acid suppression therapy significantly worsened chronic kidney disease (CKD) [90], and the European Association for the Study of the Liver (EASL) guideline also described it as well [91]. However, a prospective study showed that approximately 3 months of acid suppression therapy does not have a connection with SBP [92].

### **CQ4-5**. Which is more useful in preventing recurrence of esophageal varices (EV): endoscopic variceal ligation (EVL) or endoscopic injection sclerotherapy (EIS)?

- Both EVL and EIS are proposed for treatment.

(Recommendation: weak, 100% agreed, evidence level C).

*Comment*: A meta-analysis comparing EVL to EIS in preventing recurrence of EV showed that there is no difference in the rebleeding rate or survival rate between EVL alone and EVL combined with EIS [93, 94]. In the western countries, the first-line treatment of preventing recurrence of EV is EVL combined with NSBBs and a lot of evidence has been shown in this treatment area [91, 95, 96]. However, in a trial to prevent EV recurrence in Japan, there was significantly more post-treatment whole circumference ulcer formation in the EIS alone group than in the EIS combined with EVL group. Repeat EIS was more effective than EVL combined with EIS because the recurrence rate in the combination group was significantly higher than that in the alone group [97].

### **CQ4-6**. Is balloon-occluded retrograde transvenous obliteration (BRTO) useful for gastric varices and encephalopathy?

- We propose BRTO for the prevention of rebleeding in isolated gastric varices.
  (Recommendation: weak, 100% agreed, evidence level C).
- We also propose BRTO for hepatic encephalopathy derived from large portosystemic shunts.
  (Recommendation: weak, 100% agreed, evidence level C).

*Comment*: BRTO, developed by Kanagawa et al. [98] as a prophylactic/preventive treatment, is widely used in Japan and was covered by insurance in 2018 based on the results of an investigator-initiated clinical trial [99]. However, there are no randomized trials to demonstrate the efficacy of prophylactic treatment, and it is not recommended according to European and American guidelines [91, 96]. In Japan, Akahoshi et al. [100], although in a case–control study, showed data on the efficacy of BRTO, with improved survival in patients with a history of hemorrhage. The results of this study suggest that BRTO can be used to prevent rebleeding in gastric varices. European and American guidelines [91, 96] similarly recommend BRTO for the prevention of rebleeding, but more evidence is needed to support its effectiveness. However, there are no randomized data or systematic reviews on the efficacy of BRTO in hepatic encephalopathy, although case–control studies are available [101–104].

### **CQ4-7**. Does injection of *n*-butyl cyanoacrylate (NBCA) improve patients’ survival in the treatment for prevention of fundal gastric variceal (GV) bleeding?

- In patients who have never had GV bleeding, injection of NBCA improves the survival. However, in patients who have had GV bleeding, injection of NBCA is not proposed because BRTO is better than injection of NBCA for the prevention of fundal GV rebleeding.

(Recommendation: weak, 93% agreed, evidence level C).

*Comment*: Emergent treatment for GV bleeding is endoscopic hemostasis using cyanoacrylate (CA) [91, 96]. Mishra et al. reported [105] that the actuarial probability of non-bleeding from gastric varices over a median follow-up period of 26 months was 87% in the CA group, 72% in the NABBs group, and 55% in the non-treatment group. The actuarial probability of survival was higher in the CA group than in the no-treatment group (90% vs. 72%). However, Akahoshi et al. [100] reported that in 110 patients with GV bleeding, BRTO was superior to EIS with NBCA in the prevention of rebleeding and survival rates.

## Ascites

### **BQ 4–3**. What is useful in diagnosis of cirrhotic ascites and spontaneous bacterial peritonitis (SBP)?

**Fig. 3:**
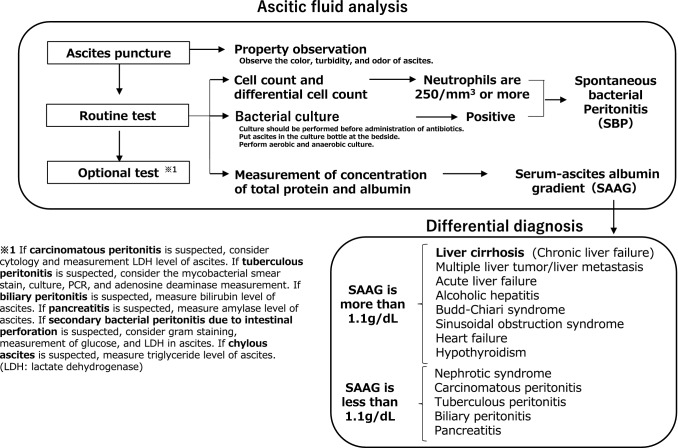
Diagnostic algorithm for cirrhotic ascites. As a routine test, ascitic fluid obtained by diagnostic paracentesis should be examined for total protein, albumin, cell count, and differential cell count. The serum–ascites albumin gradient (SAAG) is an index for estimating the cause of ascites. Ascites can be diagnosed as leaky when it is 1.1 g/dL or more and exudative when it is less than 1.1 g/dL. SAAG is more reliable than the exudate-transudate concept, defined by transudate if the protein concentration of ascites is 2.5 g/dL or less and exudative if it is 4.0 g/dL or more. However, there are exceptions to these indicators; therefore, it is necessary to make a comprehensive judgment. Spontaneous bacterial peritonitis (SBP) is diagnosed when the bacterial culture is positive or when the neutrophil count is 250/mm^3^ or higher, even if the bacterial culture is negative. The leukocyte esterase test strip is a simple and rapid diagnostic tool for SBP and is useful in situations where it is difficult to calculate the number of neutrophils

- The serum-ascites albumin gradient (SAAG) is a useful index in diagnosis of cirrhotic ascites, as SAAG ≥ 1.1 g/dL indicates that portal hypertension is involved in ascites formation with high accuracy. However, there are exceptions; therefore, it is necessary to determine the cause as a whole (Fig. 3). The combination of neutrophil count and bacterial culture in ascitic fluid is useful for the diagnosis of SBP.

### **BQ4-4**. Is salt restriction effective for ascites associated with cirrhosis?

- Appropriately limiting salt intake to a level that does not impair appetite (5–7 g/day) is effective.

### **BQ4-5**. Is albumin infusion effective for treatment of cirrhotic patients with ascites?

- In patients with hypoalbuminemia, the administration of albumin in combination with diuretics promotes the disappearance of ascites, reduces the recurrence of ascites, reduces the incidence of complications, and improves the prognosis.
- Administration of albumin during large-volume paracentesis (LVP) prevents circulatory dysfunction and improves prognosis. (See BQ4-8)
- Administration of albumin in patients with spontaneous bacterial peritonitis (SBP) or type 1 hepatorenal syndrome (HRS-AKI) is effective in improving prognosis.

### **BQ4-6**. What is the effective method of administration of spironolactone and loop diuretics for ascites in cirrhosis?

- When initiating monotherapy for ascites in cirrhosis, spironolactone should be administered as first-line therapy. If the therapeutic effect of spironolactone alone is inadequate, the combination of spironolactone and a loop diuretic is recommended to prevent the adverse effects associated with higher doses of spironolactone. The superiority of sequential addition of a loop diuretic after prior spironolactone monotherapy and initiation of the spironolactone and loop diuretic in combination as initial treatment has not been conclusively determined.

### **BQ4-7**. Are vasopressin V_2_ receptor antagonists useful for the management of cirrhotic ascites?

- A vasopressin V2 receptor antagonist in combination with conventional diuretics (spironolactone with or without furosemide) is useful for managing cirrhotic ascites.

### **BQ4-8**. Is large-volume paracentesis (LVP) useful for patients with refractory ascites?

- LVP is useful for the management of ascites [106]. Paracentesis is recommended as the first-line therapy for patients with diuretic-resistant ascites. Paracentesis-induced circulatory dysfunction (PICD) may occur in these patients; therefore, albumin infusion in combination is recommended for the prevention of PICD [107].

### **BQ4-9**. Is peritoneovenous shunt (PVS) therapy useful for patients with refractory ascites?

- In patients with refractory ascites with no other therapeutic options, PVS should be performed after cautious assessments and obtaining informed consent.

### **BQ4-10**. Does the prognosis of cirrhosis worsen with spontaneous bacterial peritonitis (SBP) or other infections?

- The prognosis can worsen. The 1-year survival rate from the onset of SBP is approximately 40%.

### **CQ4-8**. Are prophylactic antibiotics useful for severely cirrhotic patients with ascites?

- Prophylactic antibiotics are recommended depending on the risk of infection. However, prophylactic antibiotics are not covered by insurance in Japan.

(Recommendation: week, 73% agreed, evidence level B).

*Comment*: Recent international or Japanese guidelines recommend prophylactic antibiotics in patients with cirrhosis at high risk for the development of infection [1, 108–110]. Therefore, in cirrhotic patients with ascites, those with upper gastrointestinal bleeding, and those with a history of SBP, prophylactic antibiotics to inhibit the onset or relapse of SBP is recommended (prophylactic antibiotics are not covered by insurance in Japan); however, strict monitoring of the emergence of resistant bacteria is required. In a comparative study of norfloxacin vs. placebo on the emergence of resistant bacteria, 11 of the 13 g-negative bacilli isolated from the norfloxacin-treated group were resistant to quinolone antibiotics, whereas in the 6 g-negative bacilli isolated from the placebo group, only one was resistant to quinolone antibiotics (*P* = 0.01) [111]. Resistant bacteria were mainly detected in urine. No clear conclusions have been reached regarding which antibiotics are the most suitable for the prevention of infections [112, 113]. In case of improvement of clinical symptoms, such as disappearance of ascites, prophylactic antibiotics should be discontinued to prevent the emergence of resistant bacteria [111].

### **CQ4-9**. Is cell-free and concentrated ascites reinfusion therapy (CART) useful for refractory ascites associated with patients with cirrhosis?

- It may be as useful as large-volume paracentesis (LVP) with albumin infusion, and is proposed for the management of such patients.

(Recommendation: weak, 100% agreed, evidence level B).

*Comment*: The concentrated ascites reinfusion therapy, CART, was developed as a modification of LVP. This therapy aims to maintain serum albumin levels by filtrating and concentrating the removed ascitic fluid, followed by intravenous reinfusion of the collected proteins. It is as safe and effective as total paracentesis with albumin infusion for the treatment of tense ascites in patients with cirrhosis [114]. One of the benefits of CART is the reduced use of albumin, but its disadvantages include the cost of instruments and staff and allergic reactions [115].

### **CQ4-10**. When is the appropriate time to administer vasopressin V_2_ receptor antagonist for cirrhotic ascites?

- For patients with cirrhotic ascites who are resistant to conventional diuretics, it is recommended to start administration of tolvaptan at an early stage when renal function is preserved without increasing the dose of spironolactone (25–50 mg/day) with or without loop diuretics, such as furosemide (20–40 mg/day).

(Recommendation: strong, 100% agreed, evidence level B).

**Fig. 4:**
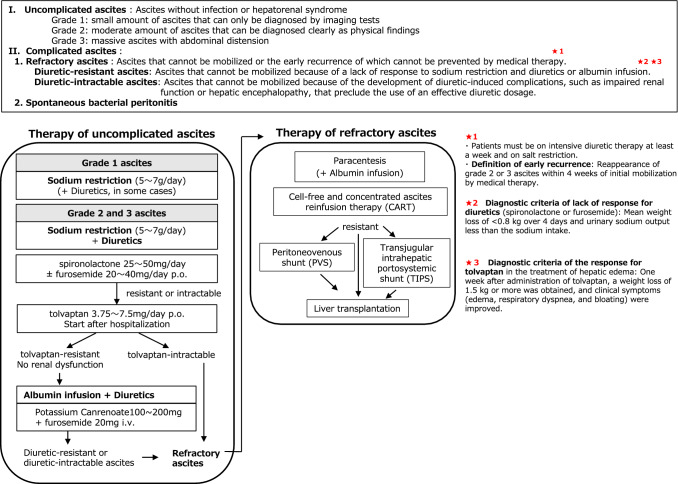
Therapeutic algorithm for cirrhotic ascites. Grade 1 ascites is treated with sodium restriction (5–7 g/day) and, in some cases, diuretics. For grade 2 and 3 ascites, spironolactone (25–50 mg/day) is administered as a first-line drug with sodium restriction. When the effect is insufficient, furosemide (20–40 mg/day) is used in combination. In cases of resistant or intractable tolvaptan (3.75–7.5 mg/day) is additionally administered after hospitalization. For tolvaptan-resistant patients without renal dysfunction, intravenous injection of potassium canrenoate (100–200 mg) and furosemide (20 mg) is started. For severe hypoalbuminemia (< 2.5 g/dL), albumin infusion is considered. For **refractory ascites**, paracentesis or cell-free and concentrated ascites reinfusion therapy (CART) is recommended. Albumin infusion in large-volume paracentesis is effective in preventing paracentesis-induced circulatory dysfunction (PICD). Peritoneovenous shunts or transjugular intrahepatic portosystemic shunt (TIPS) are recommended for resistant cases. If these treatments are ineffective, liver transplantation should be considered

*Comment*: Tolvaptan, a vasopressin V_2_ receptor antagonist, is a diuretic that inhibits the absorption of water by vasopressin in the collecting duct of the kidney and excretes only water without affecting the excretion of electrolytes. In the domestic clinical trials, the addition of tolvaptan (7.5 mg/day for 7 days) was more effective than conventional diuretics on ascites in multicenter RCTs for poor responders to standard diuretic therapy (spironolactone at ≥ 25 mg/day and furosemide at 40 mg/day or higher, or spironolactone at 50 mg/day or higher and furosemide at 20 mg/day or higher) [116, 117]. Based on these results, tolvaptan is approved in Japan as an additional drug for fluid retention in patients with liver cirrhosis in whom conventional diuretics (spironolactone with or without loop diuretics, such as furosemide) are ineffective. The disadvantage of furosemide is that high-dose administration causes renal injury and electrolyte abnormalities [106]. In addition, the complication of renal dysfunction in patients with liver cirrhosis is significantly associated with a worsening prognosis [118]. From the point of preventing deterioration of renal function, we recommend the early additional administration of tolvaptan (See Fig. 4).

### **FRQ 4–1**. Are there any factors to predict the effect of vasopressin V_2_ receptor antagonist for cirrhotic ascites?

- Factors to predict the response of tolvaptan include indicators such as BUN value and urinary sodium excretion / concentration that reflects renal function.

### **FRQ4-2**. Is transjugular intrahepatic portosystemic shunt (TIPS) useful for refractory ascites associated with patients with cirrhosis?

- TIPS is more effective than LVP with albumin infusion for the treatment of refractory ascites associated with cirrhosis in terms of both ascites control and survival, but it is also more likely to cause hepatic encephalopathy. Although improvements in stents and techniques have improved the outcome of treatment, there is no insurance coverage in Japan.

## Hepatorenal syndrome (HRS)

### **BQ4-11**. Does renal dysfunction worsen the prognosis of patients with liver cirrhosis?

- Renal dysfunction worsens the prognosis of patients with Liver cirrhosis.

### **BQ4-12**. Does liver transplantation (LT) improve the prognosis of liver and kidney syndrome?

- LT improves the prognosis for liver and kidney syndrome.

### **CQ4-11**. Are there effective drugs for HRS?

- Presently, the combined administration of noradrenaline and albumin has been suggested.
  (Recommendation: weak, 73% agreed, evidence level B)

*Comment*: The basis of treatment of HRS is the administration of vasoconstrictive agents with albumin to ensure circulating plasma volume. Regarding vasoconstrictive sympathomimetic agents, the combination therapy of noradrenaline, alpha and beta sympathomimetic agents, and furosemide has been reported to be beneficial in pilot studies [119]. Under albumin concomitant conditions, the efficacy of noradrenaline in cases with HRS was reported to be comparable to that of vasopressin’s synthetic analogue terlipressin and to be cost-effective [120–122]. It has been reported that noradrenaline is as effective as a combination therapy of octreotide, a synthetic analogue of somatostatin promoting smooth muscle contraction, and midodrine, a sympathomimetic agent in patients with HRS under albumin concomitant conditions, and is as effective as terlipressin and better than combination therapy of midodrine and octreotide for restoring kidney function [123, 124]**.**

Considering the cost performance and a long experience of using noradrenaline and the difficulty in using the other abovementioned drugs in Japan, we suggest a combination of noradrenaline and albumin for the treatment of HRS at present.

### **FRQ4-3**. Is ultrasonography useful for the diagnosis of HRS?

- Although ultrasonography is not useful for the diagnosis of HRS, doppler ultrasonography may help predict the risk of developing HRS.

### **FRQ4-4**. Are transjugular intrahepatic portosystemic shunts (TIPS) useful for the management of HRS?

- In appropriately selected patients, TIPS improves renal function, reduces ascites, and can be expected to improve prognosis [125], although TIPS is not supported by health insurance in Japan.

## Hepatic encephalopathy

### **BQ4-13**. Is non-absorbable synthetic disaccharide useful for hepatic encephalopathy?

- Nonabsorbable synthetic disaccharides are effective and should be administered as basic treatment for hepatic encephalopathy.

### **BQ4-14**. Is a branched-chain amino acid (BCAA)-related medication effective for the management of hepatic encephalopathy?

- BCAA-related medication is effective for the management of hepatic encephalopathy.

### **CQ4-12**. Is treatment necessary for covert hepatic encephalopathy?

- We suggest treating patients with covert hepatic encephalopathy who have a high risk of developing overt hepatic encephalopathy, such as worsening background liver conditions or the presence of symptoms of decompensated cirrhosis other than hepatic encephalopathy.

(Recommendation: weak, 100% agreed, evidence level B).

*Comments*: Hepatic encephalopathy is an important complication of cirrhosis, and its symptoms range from nonspecific neurological or psychological abnormalities to coma. The diagnosis of minimal hepatic encephalopathy (MHE) or covert hepatic encephalopathy (CHE) is important for prognostic estimation and evaluation of a patient’s quality of life. There are some clinical trials on MHE and CHE, but most of the trials have been conducted for less than 6 months and do not reflect the long-term course of the disease [126]. Moreover, most of them have small sample size and are open trials. Interventional methods are also heterogeneous. Some studies have reported prolongation of time to the first episode of overt hepatic encephalopathy (OHE) with synthetic disaccharide administration in a small number of cases [127]. However, the diagnosis of MHE and CHE itself is not uniform, and different interventions and assessment methods have been used [128, 129]. Currently, in western clinical guidelines, only OHEs and some CHEs are recommended to be treated [130]. In other words, MHEs or CHEs are considered for the general prophylactic treatments only if there are obvious high-risk factors (e.g., worsening background liver conditions and symptoms of decompensated cirrhosis other than hepatic encephalopathy). General prophylactic treatments for MHE and CHE should not be recommended.

### **CQ4-13**. Are non-absorbable antimicrobials useful for hepatic encephalopathy?

- Since non-absorbable antimicrobial agents are effective for hepatic encephalopathy both in initial or recurrent episode, its administration is a basic treatment for hepatic encephalopathy.

(Recommendation: strong, 100% agreed, evidence level A).

*Comment*: Intestinal non-absorbable antimicrobial agents have been used for the treatment of hepatic encephalopathy, and their mechanism of action is thought to be the inhibition of ammonia-producing bacteria in the intestine (see BQ4-13). Rifaximin improves clinical symptoms and neuropsychological symptoms in hepatic encephalopathy [131]. Rifaximin reduces the risk of recurrence of hepatic encephalopathy. This effect was also observed in CHE, as assessed by objective measures. In addition, driving abilities of patients with CHE were reportedly improved. The results of a Japanese phase III study demonstrated that rifaximin improved plasma ammonia levels and clinical parameters, such as the portal systemic encephalopathy index (PSE index) and number connection test A (NCT-A) [132]. The long-term (24 months) efficacy and safety of rifaximin in hepatic encephalopathy has also been reported [133]. In addition, a recent systematic review showed that rifaximin may prevent recurrence of hepatic encephalopathy and may also reduce mortality [134]. Other meta-analyses have reported that rifaximin administration is effective and safe, similar to non-absorbable synthetic disaccharides [135]. However, there is no high level of evidence for first-line treatment, such as nonabsorbable synthetic disaccharides. Nonetheless, there are sufficient data to support the efficacy and safety of rifaximin’s use; thus, we recommend its use as a basic treatment for hepatic encephalopathy similar to that recommended by other global guidelines [130].

### **CQ4-14**. Is zinc preparation useful for hepatic encephalopathy?

- Since zinc deficiency can often be present in patients with cirrhosis, we suggest supplementation with zinc preparations for patients with hepatic encephalopathy with possible zinc deficiency.

(Recommendation: weak, 77% agreed, evidence level B).

*Comment*: Although there have been trials involving zinc for the treatment of hepatic encephalopathy, most of them were short term. Moreover, there are very few RCTs. In addition, the long-term efficacy (e.g., recurrence rates and mortality of hepatic encephalopathy) is unknown in these older studies. However, an RCT from Japan found that 6 months of oral zinc supplementation resulted in improvements in health-related quality of life (HRQOL), plasma ammonia levels, and degree of hepatic encephalopathy, as well as improvements in Child–Pugh scores and neuropsychiatric tests [136]. No serious adverse effects associated with the administration of zinc products were reported in these studies. A recent meta-analytic systematic review demonstrated that zinc preparations resulted in a significant improvement in the NCT score, an objective measure of hepatic encephalopathy [137]. However, long-term efficacy, liver-related mortality, and quality of life changes have not been established. Furthermore, there are no clear criteria for the diagnosis of zinc deficiency in patients with cirrhosis.

### **CQ4-15**. Is carnitine supplementation useful for hepatic encephalopathy?

- Since carnitine deficiency is often present in patients with cirrhosis, we suggest carnitine supplementation for patients with hepatic encephalopathy with possible carnitine deficiency.

(Recommendation: weak, 92% agreed, evidence level B).

*Comment*: In Japan, L-carnitine is used for carnitine deficiency. However, the diagnosis of carnitine deficiency in patients with liver cirrhosis is based on clinical symptoms, which are essentially objective. Therefore, the definition of carnitine deficiency differs in various clinical trials and may thus include several biases. The administration of carnitine for hepatic encephalopathy has been shown to be effective in several RCTs ranging from short-term to medium-term studies [138]. The parameters used for evaluation also vary according to the degree of hepatic encephalopathy. In addition, the assessment items for the improvement of hepatic encephalopathy are heterogeneous; some are clinical symptoms, and others are cognitive abilities. Moreover, there are few multicenter studies. In Japan, L-carnitine supplementation was reported to reduce blood ammonia levels. A recent open randomized trial from Japan reported that 12 weeks of carnitine supplementation improved blood ammonia and NCT [139]. In addition, a recent systematic review summarized that acetyl-L-carnitine administration reduced ammonia levels and improved NCT [138]. Thus, the level of evidence for the use of carnitine in hepatic encephalopathy is increasing, although evidence for the improvement in long-term prognosis, which is an important clinical factor, has not been established.

* NCT: number connection test.

### **CQ4-16**. Are probiotics useful for hepatic encephalopathy?

- Probiotics have been reported to improve the parameters of hepatic encephalopathy in patients with mild hepatic encephalopathy.

(Evidence level C).

*Comment*: Probiotics are defined as microorganisms or drugs and foods that contain such microorganisms that are thought to have a positive effect on the human body. It is presumed that components derived from microorganisms, such as peptidoglycans, secreted proteins, and enzymes, act on intestinal bacteria, intestinal epithelial cells, and immunocompetent cells present in the intestinal mucosa. Hepatic encephalopathy is a condition in which neurotoxic substances, such as ammonia, mercaptan, and phenol, are not adequately metabolized by the liver and their levels increase in blood. Many neurotoxic substances are derived from intestinal bacteria, and the usefulness of probiotics to improve the composition of intestinal bacteria has been investigated for the treatment of encephalopathy. In a randomized trial of patients with subclinical encephalopathy, there was a significant improvement in encephalopathy parameters in the probiotic group compared to those in the control group [140]. On the contrary, a systematic review comparing probiotics with placebo or no treatment showed no significant differences in mortality from any cause, and the non-recovery and adverse event rates, including hepatic encephalopathy, were lower in the probiotic group, but the effect on hospitalization was unknown, with no significant difference between the two groups [141]. Quality of life was slightly improved in the probiotic group, and the effects of probiotics versus lactulose on mortality, non-recovery, and adverse event rates, including encephalopathy, hospitalization, and quality of life, were reported to be unknown due to the very low quality of evidence [142]. Probiotics have been reported to be more useful than placebo or no intervention in preventing or improving subclinical encephalopathy, but their effects have been reported to be comparable to those of lactulose [143, 144]. However, due to the presence of serious biases in both inclusion criteria and heterogeneity of intervention methods, the true effects of probiotics for the treatment of hepatic encephalopathy have not been established. Thus, currently, the usefulness of probiotics is not conclusive and they are not recommended for the treatment of hepatic encephalopathy, similar to other clinical guidelines [130].

## Portal vein thrombosis (PVT)

### **BQ4-15**. What is the pathogenesis and prognosis of PVT associated with cirrhosis?

- PVT occurs in 10–25% of patients with cirrhosis and may lead to an exacerbation of long-term prognosis.

### **CQ4-17**. What is an effective treatment for PVT associated with cirrhosis?

- Administration of anticoagulant is suggested with consideration of the prognostic impact of PVT.

(Recommendation: weak, 100% agreed, evidence level B).

*Comment*: Low-molecular-weight heparin or vitamin K antagonists have been used as anticoagulation therapy for PVT, but increased bleeding events have been a concern as a side effect. Two meta-analyses have shown no differences in bleeding side effects [145, 146] and have suggested that there is no need to avoid anticoagulant therapy. Recently, trials using direct oral anticoagulants (DOACs) and antithrombin III have also been reported [147–149]. However, these studies were conducted in a small number of cases. Currently, there are no drugs with strong evidence to recommend them [150].

The need for maintenance therapy after resolution of the thrombus depends on the recurrence rate after treatment cessation. Of the 81 patients treated with anticoagulants, 46 patients underwent recanalization, but 17 (36%) relapsed after treatment was discontinued [151]. Therefore, discontinuation after recanalization should be carefully decided in patients who are deemed eligible for PVT treatment.

A single-center, randomized, prospective study was reported to show the prophylactic efficacy of low-molecular-weight heparin in patients with advanced cirrhosis without PVT. A prospective comparison of 70 patients with Child-Pugh B7-C10 randomized to receive enoxaparin or no treatment has shown a significant reduction in the development of PVT and progression to liver failure and improved prognosis [152]. Validation studies by other institutions are also needed for the prophylactic use of anticoagulants.

## Sarcopenia

### **CQ4-18**. Does sarcopenia affect the prognosis of cirrhotic patients?

- Assessment for sarcopenia in cirrhotic patients is recommended because it adversely affects the prognosis.

(Recommendation: strong, 100% agreed, evidence level A).

*Comment*: Primary sarcopenia is a condition in which skeletal muscle mass and strength or physical function decline with aging, while secondary sarcopenia is defined as a condition in which skeletal muscle mass and strength or physical function are impaired due to underlying diseases [153]. In most Japanese studies on the relationship between sarcopenia and prognosis in liver diseases, sarcopenic cases are reported to have a significantly lower survival rate than non-sarcopenic cases [154]. The development of sarcopenia in patients awaiting liver transplantation overseas is reported to be strongly associated with mortality as well as a higher Model for End-Stage Liver Disease (MELD) score [155]. Sarcopenia increases the risk of cirrhosis-related complications and adversely affects prognosis [156]. Sarcopenia accompanied by an increase in fat mass (sarcopenic obesity) is also attracting attention as an adverse predictor in cirrhotic patients [157].

Several differences can be found between the sarcopenia assessment criteria for liver diseases proposed by the Japan Society of Hepatology (JSH) and those proposed in other countries [154, 158–161]. The European Working Group on Sarcopenia in Older People 2 (EWGSOP2) (revised version) recommends the use of SARC-F (strength, assistance walking, rise from a chair, climb stairs, and falls) as a screening tool for sarcopenia [159]. The Asian Working Group for Sarcopenia (AWGS) (revised version) recommends the use of measurement of calf circumference or SARC-F as a screening tool for sarcopenia [161]. In particular, according to the revised version of AWGS, sarcopenia can be diagnosed even in facilities where it is difficult to measure muscle mass [161]. The Japanese Association on Sarcopenia and Frailty recommends the use of the finger-circle test (Yubi-wakka test) as a screening tool for sarcopenia, and its usefulness has been reported in patients with liver diseases [162].

### **CQ4-19**. Is there a useful treatment for sarcopenia associated with cirrhosis?

- Exercise and nutritional therapies are proposed.

(Recommendation: weak, 92% agreed, evidence level C).

*Comment*: In an RCT by Les et al. in 116 cirrhotic patients with a history of hepatic encephalopathy, hepatic encephalopathy and skeletal muscle mass were significantly improved in the BCAA-treated group compared to that in the maltodextrin-treated group [163]. A retrospective study by Hanai et al. reported that cirrhotic patients with sarcopenia had a better prognosis in the BCAA-treated group than in the non-BCAA-treated group [164]. Improvement in sarcopenia has been reported by exercise for 8 to 14 weeks in four RCTs [165–168]. However, exercise intervention for Child-Pugh C patients cannot be recommended presently. Furthermore, the outcomes of long-term exercise in cirrhotic patients have not been clarified. Especially in elderly cirrhotic patients, the risk of falls due to exercise can increase, and exercise in cirrhotic patients with varices can lead to an elevated risk of varices rupture. The usefulness of combined exercise and leucine therapy for sarcopenia has been reported in an RCT with placebo control by Román et al. [169]. In Japan, although not in an RCT, combined exercise and BCAA therapy has been reported to improve sarcopenia [170, 171]. An animal study suggests that improvement in hyperammonemia can lead to improvement in sarcopenia [172]. A retrospective study reported that l-carnitine led to the improvement in sarcopenia through improvement of hyperammonemiareduction in hyperammonemia [173]. Regarding RCTs related to hormone replacement therapy, only one volume reported the usefulness of testosterone therapy for male cirrhotic patients [174].

## Muscle cramps

### **CQ4-20**. Is there a useful treatment for muscle cramps associated with cirrhosis?

- Shakuyaku-kanzo-to, l-carnitine, BCAA, and zinc are proposed according to the pathological condition.

(Recommendation: weak, 80% agreed, evidence level C).

*Comment*: In Japan, shakuyaku-kanzo-to, l-carnitine, BCAA, zinc, etc., have been commonly used for cirrhosis-related muscle cramps [175–179]. A prospective study by Nakanishi et al. reported that patients with cirrhosis (*n *= 23) who received 1200 mg of l-carnitine daily had a significantly higher rate of disappearance of muscle cramps than those (*n *= 19) who received 900 mg of l-carnitine daily (43.5% vs. 10.5%), and improvement in muscle cramps was observed in 80% or more out of the 42 cases [176]. Hidaka et al. reported that in an RCT of 37 cirrhotic patients, the frequency of lower extremity muscle cramps was significantly lower in the patients who received BCAA granules before bedtime than in those who received during daytime [177]. In a prospective study by Hiraoka et al., in 18 cirrhotic patients receiving BCAAs, l-carnitine (1000 mg/day) and additional exercise (2000 steps/day) significantly improved muscle cramps [178]. In addition, a previous study reported that BCAA as late evening snacks was useful for cirrhosis-related muscle cramps [179]. In a cohort study by Hiraoka et al. in 289 cirrhotic patients, 160 (55.4%) had muscle cramps, and in 82 patients treated with any medicine, l-carnitine was most frequently used (66 cases, 80.5%) and pharmacological intervention for muscle cramps significantly improved the quality of life [175]. In a cohort study of 432 patients with liver disease, 112 (25.9%) had muscle cramps, and female sex, diabetes, and renal disease were significant predictors of muscle cramps [180].

## Others

### **CQ4-21**. Is thrombopoietin receptor agonist effective for thrombocytopenia in patients with liver cirrhosis?

- Treatment with thrombopoietin receptor agonist is recommended for thrombocytopenia in patients with liver cirrhosis prior to elective invasive procedures.

(Recommendation: strong, 100% agreed, evidence level B).

*Comment*: Three RCTs have demonstrated that treatment with lusutrombopag, a thrombopoietin receptor agonist, significantly increased platelet count and reduced the need for platelet transfusions in patients with thrombocytopenia undergoing invasive procedures [181–183]. In a phase 3 clinical trial, lusutrombopag-related adverse events were reported in 8.3% (4/48) of patients, including PVT. A meta-analysis has reported that treatment with thrombopoietin receptor agonists was not associated with PVT in patients with chronic liver disease [184]. However, cases of thrombosis have been reported in clinical trials [181–183]; therefore, we must pay attention to the development of thrombosis during and after treatment with thrombopoietin receptor agonists.

### **CQ4-22**. Is the oral anti-pruritus agent, nalfurafine hydrochloride, effective for pruritus in patients with chronic liver disease?

- Treatment with nalfurafine hydrochloride, an oral anti-pruritus agent, is recommended for pruritus in patients with chronic liver disease.

(Recommendation: weak, 100% agreed, evidence level B).

*Comment*: The prevalence of pruritus has been reported to be 40.3%, and antihistamine agents or antiallergic agents have been shown to be insufficient to suppress pruritus in patients with chronic liver disease [185]. An RCT has demonstrated that nalfurafine hydrochloride, a selective κ-opioid receptor agonist, significantly improved refractory pruritus with no clinically significant adverse drug reactions in patients with chronic liver disease [186]. A case series study showed that nalfurafine hydrochloride suppressed pruritus for more than 20 weeks with no significant safety problems [187]. A prospective study has shown high recurrence rates of pruritus after the cessation of nalfurafine hydrochloride in patients with chronic liver disease [188]. The beneficial effect of nalfurafine hydrochloride was evident in patients with primary biliary cholangitis [186, 189].

### **FRQ4-5**: Are splenectomy and partial splenic embolization (PSE) effective for the improvement in the pathophysiology of liver cirrhosis?

- Splenectomy and PSE may improve the pathophysiology of liver cirrhosis. However, close attention should be paid to procedure-related complications.

### **FRQ4-6**. What is hepato-pulmonary syndrome (HPS)?

- HPS is a disorder in pulmonary oxygenation occurring in cirrhotic patients with portal hypertension. The prognosis of patients with HPS is poor, and there is currently no established pharmacotherapy for HPS.

### **FRQ4-7**. What is portopulmonary hypertension (PoPH)?

- PoPH is a type of pulmonary arterial hypertension associated with portal hypertension. The prognosis of patients with PoPH is poor if it is not properly diagnosed and treated. The therapeutic strategy for PoPH is based on the strategy for pulmonary arterial hypertension; however, this is a subject for future research.

### **FRQ4-8**. Does vitamin D deficiency affect the pathophysiology and prognosis of patients with cirrhosis?

- Vitamin D deficiency may be involved in disease progression and poor prognosis in patients with liver cirrhosis. Further evidence is needed to determine whether vitamin D supplementation improves the prognosis and quality of life in patients with liver cirrhosis.

## Predicting prognosis

### **BQ5-1**. Are Child-Pugh classification and MELD score (MELD-Na score) useful for predicting the prognosis of patients with liver cirrhosis?

- Child-Pugh classification is useful for predicting the prognosis of patients with liver cirrhosis [190]. The MELD score is useful for predicting the short-term prognosis of patients with decompensated cirrhosis [191, 192].

### **CQ5-1**. What are the useful factors for predicting the prognosis in patients with cirrhosis except for Child-Pugh classification and MELD score (MELD-Na score)?

- Evaluation of the presence of renal dysfunction, infections, and hyponatremia are strongly recommended.

(Recommendation: strong, 100% agreed, evidence level A).

*Comment*: In patients with cirrhosis, complications of renal failure, such as hepatorenal syndrome, lead to a 7.6-fold increase in mortality [118], and infections, such as spontaneous bacterial peritonitis and sepsis, lead to a 3.75-fold increase in mortality [193]. Hyponatremia is associated with increased mortality and complications [194]. Hyponatremia is a prognostic predictor independent of the MELD score in patients waiting for liver transplantation [195].

## Liver transplant

### **BQ6-01**. Does liver transplantation improve the survival of patients with decompensated cirrhosis?

- Liver transplantation improves the prognosis of decompensated cirrhosis with an increase in the MELD score.

### **CQ6-1**. Is antiviral therapy useful in controlling HBV infection after liver transplantation?

- Since antiviral therapy efficiently controls hepatitis B viral loads after liver transplantation, it is useful and strongly recommended.

(Recommendation: strong, 100% agreed, evidence level A).

*Comment*: The control of HBV infection after transplantation is a significant factor for the prognosis of cirrhosis related to HBV. A multicenter study before the introduction of antiviral therapy for HBV found that after liver transplantation for HBV cirrhosis, survival rates after transplantation were significantly lower in cases of hepatitis B recurrence compared to overall survival [196]. Nucleos(t)ide analogs with intravenous injection of HB human immunoglobulin (HBIG) prior to liver transplantation suppress the growth of HBV before liver transplantation. Perioperative administration of HBIG in combination with long-term lamivudine showed a marked efficacy with a 1-year survival rate of 93% and 0% reinfection rate [197]. The combination of HBIG with lamivudine and other NAs resulted in nearly 100% control of HBV re-infection after liver transplantation [198–202]. Post-transplantation administration of entecavir or tenofovir, which are less likely to cause viral resistance mutations, has been shown to be useful in controlling HBV reinfection [203].

Recently, a 5.2-fold increase in the risk of HBV reinfection was reported after liver transplantation for type B cirrhosis when HBIG was discontinued in patients treated long term with a combination of HBIG and a nucleotide analogue compared to those who continued to receive HBIG [204]. In Japan, a similarly high rate of protection against HBV reinfection was achieved, and prolonged administration of HBIG has been reported as a way to further increase it [205, 206].

Recently, in liver transplantation cases from HBs antigen-negative or HBc antibody-positive donors, de novo hepatitis B has been found, and some cases had fulminant hepatitis with poor prognosis. To prevent this, the US guidelines recommend prophylactic administration of HBIG for 6–12 months after liver transplantation to recipients of liver transplants from HBc antibody-positive individuals, and Japanese guidelines recommend HBIG administration to reduced HBs antibody titers to 200 IU/L for approximately 1 year after transplantation and to 100 IU/L thereafter [207, 208].

### **CQ6-2**. Should we use antiviral therapy before and after liver transplantation for patients with type C cirrhosis?

- Antiviral therapy for hepatitis C recurrence after liver transplantation is useful and recommended.

(Recommendation: strong, 100% agreed, evidence level A).

- Antiviral therapy before liver transplantation may improve liver function, but the long-term prognosis is unclear.

(Evidence level C).

*Comment*: (1) Antiviral therapy after liver transplantation for type C cirrhosis.

Hepatitis C recurrence occurs in more than 80% of patients, directly related to graft liver survival and patient prognosis [209]. Therefore, antiviral therapy for hepatitis C recurrence is essential. Sofosbuvir/ledipasvir [210] and grazoprevir/elbasvir [211] for genotype 1 and glecaprevir/pibrentasvir [212] for pan-genotype Hepatitis C virus (HCV), which are available in Japan, are all highly effective and can be safely administered.

(2) Antiviral therapy before liver transplantation for type C cirrhosis.

The advantages of antiviral treatment before liver transplantation include improvement in liver function, which may eliminate the need for liver transplantation; prevention of HCV reinfection after liver transplantation; and treatment of patients who are unable to undergo liver transplantation. The disadvantages include the possibility of missing the opportunity for liver transplantation, lack of quality of life improvement in some cases, and residual risk of high carcinogenicity.

DAA therapy in patients registered for liver transplantation may improve liver function [213], but the long-term prognosis, quality of life, and risk of carcinogenesis after viral elimination are not clear. In 2019, sofosbuvir/velpatasvir was approved in Japan for the treatment of decompensated cirrhosis. However, the number of Child-Pugh C cases in this study was small and did not include Child-Pugh score ≥13 [43]. A hepatologist’s careful induction is recommended when administering this treatment since a death case has been reported.

### **CQ6-3**. Is liver transplantation useful for non-viral liver cirrhosis?

- Liver transplantation for non-viral liver cirrhosis is useful and should be considered.

(Recommendation: strong, 100% agreed, evidence level B).

*Comment*: In Japan, 9,245 liver transplant cases were performed between 1992 and 2017, but living-donor liver transplantation was still the majority (8,795 cases). Five-year patient survival rates after deceased-donor/living-donor liver transplantation were no-detection /77.1% for NASH, 70.5%/78.4% for alcohol, 90.9%/78.6% for AIH, 90.4%/79.3% for PBC, and 96.7%/73.3% for PSC, which were comparable liver transplant outcomes for viral cirrhosis [214].

The 1-year survival rates of patients in the Japanese liver transplant registry with Child Pugh C were 57.1% for NASH, 59.9% for alcohol, 63.5% for AIH, 39.8% for PBC, and 58.8% for PSC, with an abysmal prognosis [215]. Liver transplantation is useful even in non-viral cirrhosis with no other effective treatment options at the time of liver transplantation.

## Appendix

The members of the Guidelines Committee who created and evaluated the Japanese Society of Gastroenterology and the Japan Society of Hepatology ‘‘Evidence-based clinical practice guidelines for liver cirrhosis’’ are listed below.

## Creation Committee

Chair: Hitoshi Yoshiji (Department of Gastroenterology, Nara Medical University). Vice-chair: Sumiko Nagoshi (Department of Gastroenterology and Hepatology, Faculty of Medicine, Saitama Medical University). Members: Takemi Akahane (Department of Gastroenterology, Nara Medical University), Yoshinari Asaoka (Department of Medicine, Teikyo University School of Medicine), Yoshiyuki Ueno (Department of Gastroenterology, Yamagata University Faculty of Medicine), Koji Ogawa (Department of Gastroenterology and Hepatology, Graduate School of Medicine, Hokkaido University), Takumi Kawaguchi (Division of Gastroenterology, Department of Medicine, Kurume University School of Medicine), Masayuki Kurosaki (Department of Gastroenterology and Hepatology, Musashino Red Cross Hospital), Isao Sakaida (Department of Gastroenterology and Hepatology, Yamaguchi University Graduate School of Medicine), Masahito Shimizu (Department of Gastroenterology, Gifu University Graduate School of Medicine), Makiko Taniai (Internal Medicine, Institute of Gastroenterology, Tokyo Women's Medical University), Shuji Terai (Department of Gastroenterology, Niigata University Graduate School of Medical and Dental Sciences), Hiroki Nishikawa (Department of Hepatobiliary-Pancreatic Disease, Hyogo College of Medicine), Yoichi Hiasa (Department of Gastroenterology and Metabology, Ehime University Graduate School of Medicine), and Hisashi Hidaka (Department of Gastroenterology, Internal Medicine, Kitasato University School of Medicine).

## Evaluation Committee

Chair: Tetsuo Takehara (Department of Gastroenterology and Hepatology, Osaka University Graduate School of Medicine). Vice-chair: Satoshi Mochida (Department of Gastroenterology and Hepatology, Faculty of Medicine, Saitama Medical University). Members: Hidetsugu Saito (Division of Pharmacotherapeutics, Keio University Faculty of Pharmacy), and Katsutoshi Tokushige (Department of Internal Medicine, Institute of Gastroenterology, Tokyo Women's Medical University).

## The Japanese Society of Gastroenterology

President: Kazuhiko Koike (Graduate School of Medicine, Department of Gastroenterology, The University of Tokyo). Past President: Tooru Shimosegawa (South Miyagi Medical Center). Director Responsible: Hiroto Miwa (Division of Gastroenterology, Department of Internal Medicine, Hyogo College of Medicine), Nobuyuki Enomoto (First Department of Internal Medicine, Faculty of Medicine, University of Yamanashi).

## The Japan Society of Hepatology

President: Tetsuo Takehara (Department of Gastroenterology and Hepatology, Osaka University Graduate School of Medicine). Past President: Kazuhiko Koike (Graduate School of Medicine, Department of Gastroenterology, The University of Tokyo). Director Responsible: Kazuaki Chayama (Department of Gastroenterology and Metabolism, Graduate School of Biomedical Sciences, Hiroshima University).
